# AI-Driven Resilient Fault Diagnosis of Bearings in Rotating Machinery

**DOI:** 10.3390/s25227092

**Published:** 2025-11-20

**Authors:** Syed Muhammad Wasi ul Hassan Naqvi, Arsalan Arif, Asif Khan, Fazail Bangash, Ghulam Jawad Sirewal, Bin Huang

**Affiliations:** 1 Faculty of Mechanical Engineering, Ghulam Ishaq Khan Institute of Engineering Sciences and Technology, Topi 23640, Pakistan; swasii.naqvi@gmail.com (S.M.W.u.H.N.); fazailbangash1@gmail.com (F.B.); 2 Mechanical Engineering Department, Physical Science and Engineering Division, King Abdullah University of Science and Technology (KAUST), Thuwal 23955-6900, Saudi Arabia; 3 Department of Electrical Engineering Technology, The Benazir Bhutto Shaheed University of Technology and Skill Development, Khairpur 66020, Pakistan; jawadsirewal1@gmail.com; 4 Piezoelectric Device Laboratory, Faculty of Mechanical Engineering & Mechanics, Ningbo University, Ningbo 315211, China; huangbin@nbu.edu.cn

**Keywords:** bearing fault diagnosis, artificial intelligence, machine learning, deep learning, continuous wavelet transform (CWT), convolutional neural networks, scalograms

## Abstract

Predictive maintenance is increasingly important in rotating machinery to prevent unexpected failures, reduce downtime, and improve operational efficiency. This study compares the efficacy of traditional machine learning (ML) and deep learning (DL) techniques in diagnosing bearing faults under varying load and speed conditions. Two classification tasks were conducted: a simpler three-class task that distinguishes healthy bearings, inner race faults, and outer race faults, and a more complex nine-class task that includes faults of varying severity in the inner and outer races. In this study, the machine learning algorithm ensemble bagged trees, achieved maximum accuracies of 93.04% for the three-class and 87.13% for the nine-class classifications, followed by neural network, SVM, KNN, decision tree, and other algorithms. For deep learning, the CNN model, trained on scalograms (time–frequency images generated by continuous wavelet transform), demonstrated superior performance, reaching up to 100% accuracy in both classification tasks after six training epochs for the nine-class classifications. While CNNs take longer training time, their superior accuracy and capability to automatically extract complex features make the investment worthwhile. Consequently, the results demonstrate that the CNN model trained on CWT-based scalogram images achieved remarkably high classification accuracy, confirming that deep learning methods can outperform traditional ML algorithms in handling complex, non-linear, and dynamic diagnostic scenarios.

## 1. Introduction

**Figure 1:**
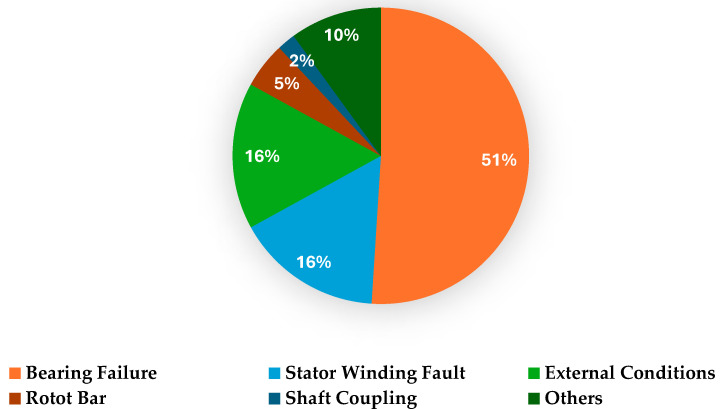
Faults in rotating machinery.

Rotating machinery plays a vital role in industries like power generation, manufacturing, transportation, aerospace, and processing industries, where they convert energy into mechanical work to perform important operations [1,2]. Rotating machinery comprises machinery that can perform tasks with the aid of rotary motions [3]. It includes various machines like turbines, compressors, pumps, and engines [4]. The performance, reliability, and efficiency of rotating machinery influence the productivity and safety of industrial processes [5]. Load fluctuation, mechanical stresses, improper maintenance, and gradual degradation over time cause defects such as mass unbalance, shaft misalignment, cracked shafts, bearing defects, and gear faults in rotating machinery [1]. These faults reduce efficiency and cause unscheduled downtimes, resulting in a loss of production and financial income [6]. A distribution of commonly occurring defects in rotating machinery is depicted in Figure 1 [7].

However, rolling bearing is one of the most essential elements in rotating equipment [8], such as rope conveyors, motors, bicycles, rolling mills, turbines, and others [9]. Bearings provide support to the ends of the rotating shaft [10]. Many studies have shown that 45–55% of overall rotating machinery failures are directly due to rolling bearing faults [11,12,13]. These faults include cracks, holes, pitting, and scratches [14]. The common causes of defects in bearings are misalignment, harsh environmental conditions, mishandling, contamination, voltage fluctuation, lubrication troubles, corrosion, and overloading [15]. If not detected in time, the faulty bearings can lead to catastrophic failure, resulting in unplanned shutdown and personnel injuries. For the secure and reliable performance of rotating machinery, it is imperative to promptly detect, isolate, and quantify the presence of defects in its bearings.

Recently, extensive research has been conducted to accurately diagnose and identify problems in rolling element bearings [16]. In the presence of defects, the system shows symptoms, like vibration, an increase in temperature, variations in the current signal, and changes in the electromagnetic field [17]. Observing and mapping these symptoms to proactively assess and predict maintenance requirements constitute the well-known framework of condition monitoring (CM) [18,19]. Condition monitoring is a maintenance technique to assess the need for maintenance before any failure occurs. It monitors machine vibration, overheating, and current signals to determine machine health [12]. Previously, time-based maintenance (TBM) was common, where maintenance was carried out based on a calendar schedule without knowing the current state of the machine. It leads to a waste of workforce, money, time, and unnecessary shutdowns [20]. A Swedish paper mill case study showed that profits can increase up to 0.975 million USD by avoiding unplanned shutdowns [21]. For this reason, CM has drawn the attention of researchers over the past few decades to prevent adverse outcomes and monetary losses [22].

In the literature, commonly employed diagnostic methods are based on the analysis of vibration [11,23,24], motor current signature [12,25], and acoustic emission (AE) data [26]. Among these methods, 90% of machines faults can be detected through vibration-based condition monitoring, as each system has a natural frequency associated with its operating conditions. Flaws or defects in a component create other dynamic loads that create oscillations in specific frequency bands [27].

Early detection of anomalies in machines can be beneficial for on-time machine maintenance. However, humans cannot respond effectively and efficiently to vast amounts of measurement data. Thus, to avoid human dependency, an intelligent condition-monitoring system should be developed [28]. During a system shutdown, almost 80% of the time is spent locating the source of the fault [29]. In recent years, artificial intelligence (AI) has gained popularity for efficient and effective bearing fault diagnosis. Data-driven bearing fault diagnosis comprises three main steps: data acquisition (DA), feature extraction, and classification [30] as per ISO 13374.

Various AI techniques for bearing fault detection, such as pattern recognition, neural networks (NNs) [31], support vector machines (SVMs) [12], K-nearest neighbors (KNNs), deep neural network, and many others [32,33,34], are used. Sawaqed et al. [35] used an artificial neural network (ANN) for the classification of vibration and motor current signals of different bearings under varying minimal operating conditions. The data for this study were provided by Paderborn University [36]. Genetic algorithms (GAs) are used to select optimal features from various time domain, frequency domain, and time–frequency domain features. The proposed method showed that the hybrid system of ANN and GA resulted in above 90% performance accuracy for both the vibration and motor current signals. Chen et al. [13] used a convolutional neural network (CNN) to extract important features from vibration signals obtained from ten different bearings at constant load, and they employed long short-term memory (LSTM) networks as a classifier. The suggested model works well in noisy environments and achieves 98.46% accuracy. Jin et al. [8] proposed that an anti-noise multi-scale convolutional neural network (AMCNN) works well in noisy environments. This model achieved 100% classification accuracy when studying constant load vibration signals of different bearings with rolling element fault, inner race fault, outer race fault, and no fault in a noisy environment. Pule et al. [37] used principal component analysis (PCA) for reducing data dimensionally and SVM as a classifier for constant load and varying speed vibration signals from eight different bearing samples, including single elements like rolling element, inner race, outer race fault, or combined faults, in which more than one element fault occurs at the same time. The classification accuracy of the suggested technique was 97.4%. Thus, this method is a good approach for variable speed conditions. Liu et al. [38] generated spectrograms through short-time fast Fourier transform (STFT) using sound signals from healthy, inner race fault, outer race fault, and rolling element fault bearing at a constant speed of 1200 RPM. Here, a deep learning model of a stacked sparse encoder algorithm was used to extract important features, and SoftMax regression was used as a classifier. The average categorization accuracy of the suggested approach was more than 95%. Furthermore, Hasan et al. [39] proposed an explainable artificial intelligence (XAI) for bearing fault diagnosis of two datasets, one from an in-house dataset at constant load and varying speed, and another dataset on bearing from Case Western Reserve University (CWRU) [40], which was obtained at constant load and varying speed conditions. The author suggested a five-stage scheme: vibration signal data preprocessing, feature extraction, feature selection, a filtration method with feature selection to avoid multicollinearity, and the use of KNN for diagnosing health conditions. The average classification accuracy for the CWRU dataset was 100%, and for the in-house dataset, it was 97%. Kankar et al. [41] studied vibration signals of ball bearings at various speeds. They employed an artificial neural network (ANN) and an SVM to classify rolling elements with corrosion pitting, outer race crack bearings, rough inner race bearing surfaces, combined bearing element defects, and healthy bearings. The results from the SVM model were relatively better than those of ANN. Pandarakone et al. [12] used current signals at a constant speed to train several deep learning and machine learning models for classifying healthy bearings from those with holes and scratches. For classifying between healthy and scratch, machine learning models of K-NN and SVM took priority with an accuracy of 83.04% and 87.85%, respectively. For deep learning, CNN showed an accuracy of 89.26%. Gao et al. [42] developed an ultra-high-speed hybrid ceramic triboelectric bearing (US-HCTEB) capable of real-time monitoring of bearing dynamics and stability. Using a crown-shaped cage and a triboelectric nano-generator mechanism, the bearing converts motion into electrical signals for condition assessment. It demonstrated excellent durability and performance up to 16,000 rpm, with dry lubrication and low humidity enhancing output. The US-HCTEB proved reliable and precise, paving the way for smart bearing applications in high-speed machinery. Guo et al. [43] proposed a novel approach using an adaptive deep convolutional neural network (ADCNN) to classify faulty and healthy bearing data from the CWRU dataset, obtained under constant load and varying speed conditions. The proposed ADCNN models showed better results compared to traditional deep convolutional neural networks (DCNNs). Deep learning requires a large dataset for accurate results. So, Yuan et al. [44] proposed an approach using CNN for feature extraction and SVM as a classifier to distinguish between vibration signals from healthy and faulty bearings. This was applied to two datasets: the CWRU bearing dataset and the Mechanical Failure Prevention Technology (MFPT) bearing dataset [45] under varying loading conditions. The average classification accuracy for the CNN-SVM-based hybrid system is 98.89%. Saucedo-Dorantes et al. [46] proposed a data-driven fault diagnosis method using deep feature learning to identify bearing faults in metallic, hybrid, and ceramic bearings. The approach employs a stacked autoencoder to automatically extract meaningful features from vibration signals in time, frequency, and time–frequency domains, followed by a feature fusion stage and a SoftMax classifier for final fault detection. A genetic algorithm was used to optimize model parameters, enhancing generalization and reducing overfitting. Tested on both a custom experimental setup and the CWRU dataset, the method achieved up to 99.8% test accuracy, demonstrating strong adaptability, robustness, and noise resistance across different bearing technologies and operating conditions. Magar et al. [47] used the CWRU and Paderborn University bearing dataset to develop a FaultNet and CNN-based model to differentiate between faulty and healthy bearings. The model showed 99% classification accuracy. Chen et al. [48] used gradient-class activation mapping (Grad-CAM) for feature selection and used a convolutional neural network (CNN) as a classifier when studying vibration signals of different bearing samples at varying speeds and constant load. Both training and testing accuracy of the proposed model are 100%.

Although the published literature showed acceptable accuracies for various fault scenarios in bearings, the following research gaps are identified:

- The majority of the algorithms focus on considering scenarios with constant speed and varying load or vice versa, often ignoring the complexity of simultaneous variation in both parameters.
- Most of the prior research work is based on small datasets that have either no or limited severity levels. For robust and generalized models, diverse high-severity levels and sufficient data must be considered.

To overcome the above-mentioned limitations, this work developed an in-house experimental setup that allows for changing the operating speeds and loading conditions for bearings in both healthy and faulty states. The time domain data was acquired from the variable operating conditions of the bearing using a low-cost three-axis accelerometer (ADXL345). Initially, the raw time domain data was processed to extract statistical discriminative features in frequency, time, and time–frequency. The ReliefF algorithm [49] was employed to order the predictors’ significance for feature selection. The performance of various machine learning algorithms (ensemble, SVM, KNN, neural network (NN), decision tree, and others) was assessed on the optimum features for distinguishing different states of the bearings. As machine learning models were not performing up to the mark, the performance of autonomous learning via a deep learning model (CNN) was assessed for the problem. In general, the deep learning models were able to accommodate the variability in speed, loading conditions, and different severity levels of the faulty states, resulting in a robust fault diagnosis model. This research paves the way for a bearing fault diagnosis system that can operate under complex conditions, including load and speed variations and varying defect severities, using artificial intelligence. This ultimately leads to reduced downtime and improved performance of rotating machines.

This paper’s remaining sections are arranged as follows: A schematic of the general technique used in the current work is given in Section 2. The philosophy behind the AI models employed in this work is described in Section 3. The experimental setup and data collection procedure are described in detail in Section 4, and the results and discussion are presented in Section 5, where the results are evaluated and contrasted with earlier research. Section 6 wraps up the work by outlining the main points, talking about any possible drawbacks, and recommending directions for further study.

## 2. Proposed Methodology

**Figure 2:**
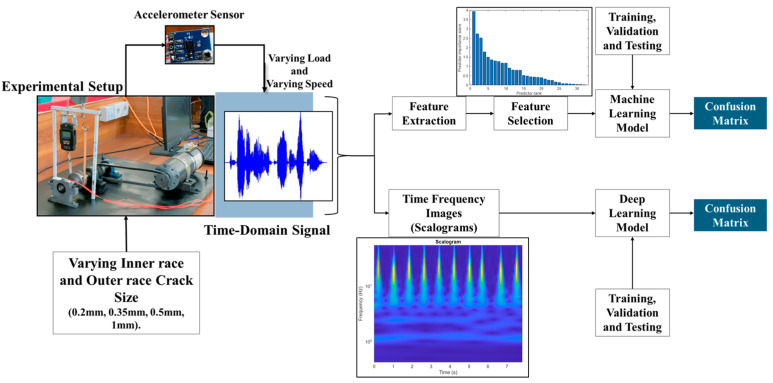
Overall methodology for fault diagnosis of bearings using machine learning and deep learning approaches.

Figure 2 shows the overall methodology for the present study. The raw vibration time domain data extracted using an accelerometer sensor from the experimental setup serves as the starting point for both deep learning and machine learning methods. In machine learning, manual feature extraction is performed, followed by feature selection. In contrast, deep learning uses neural networks to automatically extract useful information from the time–frequency images generated from the data. Both the deep learning and machine learning techniques are trained and validated. Validation is performed to optimize the model parameters accordingly. The optimized model is then tested, and a confusion matrix is plotted to visualize the model predictions.

## 3. Theoretical Background

The following section provides the theoretical background of various machine learning models that are employed in this study.

### 3.1. Ensemble Bagged Trees Model

**Figure 3:**
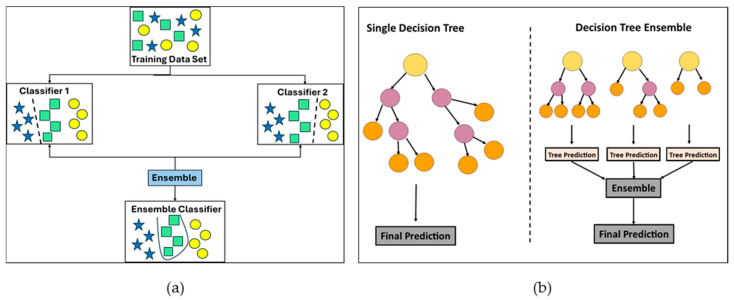
(**a**) Ensemble learning illustration example. (**b**) Ensemble bagged tree illustration example.

Ensemble learning [50] is a machine learning model that blends two or more learning techniques for better predictions, as shown in Figure 3a. The ensemble bagged trees method is a machine learning technique that reduces overfitting and increases the prediction accuracy of a model by combining many decision trees. Each decision tree is trained using a random subset of training data, as indicated by the term “bagging.” When the individual trees are trained, their predictions are combined. The bagged tree algorithm yields better stability, accuracy, lower variance, and lower bias results for complicated datasets. Figure 3b shows an ensemble bagged trees model illustration.

### 3.2. K-Nearest Neighbors

**Figure 4:**
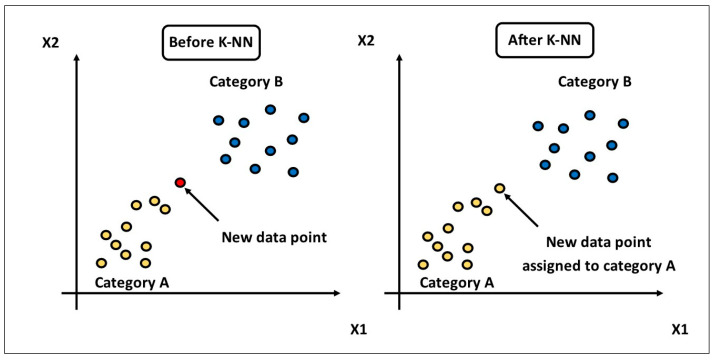
K-nearest neighbor (KNN) classification example.

For classification and regression applications, K-Nearest Neighbors (KNNs) is a straightforward, non-parametric supervised machine learning technique [51]. It works by selecting ‘K’ nearest data points or neighbors to the input, determined using a distance measure, such as Euclidean distance. In the classification problem, this means the input is assigned to the most common class, considering the neighbors. In the regression problem, the output will be the mean value of the neighbors. KNN is easy to understand and interpret; however, it is computationally costly, especially with large amounts of data, as it involves computing the difference between the new dataset and all the other datasets. Figure 4 illustrates how the KNN algorithm performs classification.

### 3.3. Support Vector Machine (SVM)

**Figure 5:**
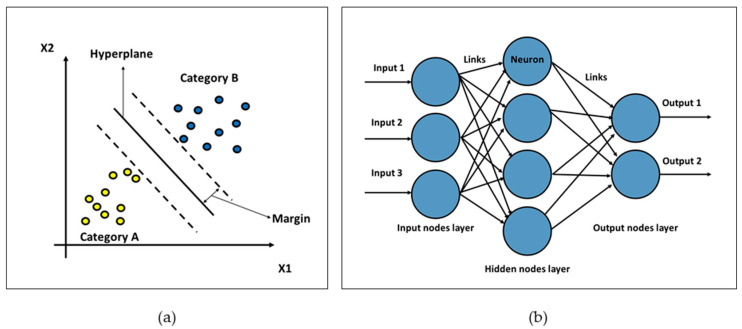
(**a**) SVM classification example. (**b**) Neural network structure for classification example.

One popular supervised machine learning approach for regression and classification is called the support vector machine (SVM). It involves finding the ideal hyperplane to divide data points into distinct classes [52]. A hyperplane is a line in two-dimensional space, but a plane in higher dimensions. To aid in model generalization, SVM seeks to optimize the margin between the nearest data points (support vectors) of each class and the hyperplane. SVM handles non-linear separable data using kernel functions. It is extensively utilized in bioinformatics, text classification, and picture recognition. Figure 5a shows how SVM works.

### 3.4. Neural Network

A neural network is an ML technique that includes interconnected nodes, known as neurons arranged in layers. Each neuron transforms the input data by applying weights, biases, and an activation function to capture non-linear patterns, passing the results to the next layers. Neural networks are thus robust for applications such as natural language processing, picture recognition, and complex function approximation. They are trained with backpropagation, which essentially involves reducing error in the network through gradient descent. Neural networks include a large number of dense hidden layers that enable models to learn important features of the data. Neural networks are widely used for classification, regression, and other prediction tasks in various fields. Figure 5b shows a basic neural network structure.

### 3.5. K-Fold Cross-Validation

**Figure 6:**
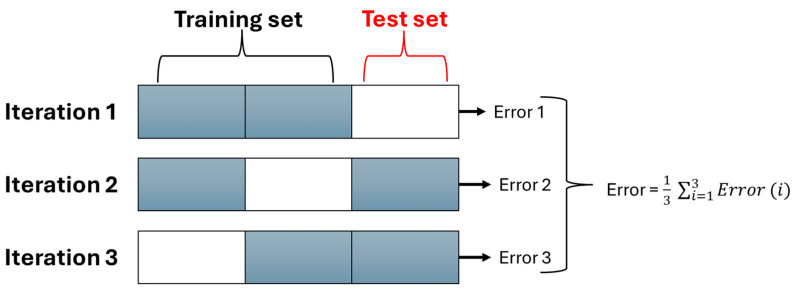
K-fold cross-validation.

K-fold cross-validation divides the dataset into “K equal parts” to assess the performance of the machine learning model [53]. The remaining “K-Fold” is used to validate the correctness of the training process once the model has been trained on “K-1 Folds.” Each K-fold is validated by repeating this process K times. It provides a trustworthy performance evaluation and aids in lowering overfitting. It is frequently employed to assess a model’s ability to generalize new data. Figure 6 shows the process of K-fold cross-validation.

The following section provides the theoretical background of deep learning models employed in this study.

### 3.6. Convolutional Neural Network (CNN)

**Figure 7:**
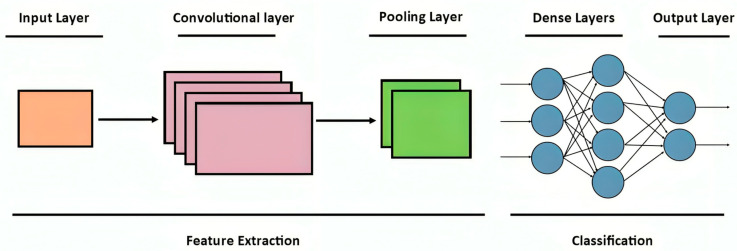
Basic CNN architecture.

A DL approach, known as a convolutional neural network (CNN), was created especially for processing grid-based data structures, such as pictures. CNN automatically learns important spatial features through convolutional layers, by applying filters to detect patterns like edges, textures, and other complex features. A pooling layer is employed to make the model computationally efficient by reducing spatial dimensions while preserving important information. Fully connected layers at the end of the layout receive feature information and make predictions with it. CNNs are widely used for image classification, object recognition, and video analysis because of their ability to detect spatial relationships and their robust performance with minimal preprocessing. It has been demonstrated that CNNs are well-suited for learning features from rotating mechanical signals due to their strength in handling periodic patterns. CNNs have therefore been effectively used by several studies to diagnose faults in rotating machinery [52]. Figure 7 shows the basic CNN architecture.

The CNN architecture used in this study was designed to achieve a balance between model simplicity, interpretability, and classification accuracy. A shallow configuration with a limited number of convolutional and pooling layers was selected after iterative experimentation, as deeper networks did not significantly improve performance but increased computational cost and risk of overfitting. The filter sizes and feature map counts were chosen based on their ability to capture both localized time patterns and broader frequency structures from the scalogram images. This configuration ensured stable training behavior, consistent convergence, and higher accuracy across all fault categories, making it an efficient and reproducible framework for vibration-based fault diagnosis.

### 3.7. Continuous Wavelet Transform (CWT)

**Figure 8:**
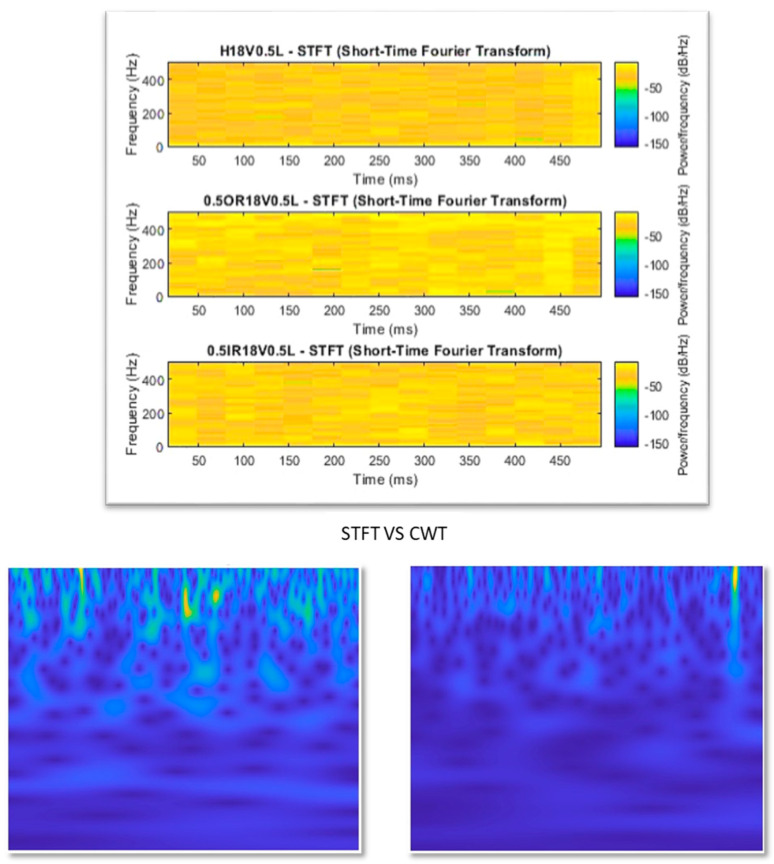
STFT vs. CWT for different classes at the same condition.

The continuous wavelet transform (CWT) is a powerful and flexible technique for analyzing nonstationary vibration signals, making it particularly suitable for mechanical fault diagnosis. It decomposes signals into small wavelets localized in both time and frequency, enabling the detection of transient and scale-dependent features that conventional methods often overlook [54]. Traditional time domain analysis can obscure important frequency information, while frequency domain techniques such as the fast Fourier transform (FFT) lose all temporal resolution. The short-time Fourier transform (STFT) partially addresses this issue. However, its fixed window size limits adaptability. Small windows offer better time resolution at the cost of frequency accuracy, while large windows do the reverse [55]. In contrast, CWT employs variable window sizes, which are narrow at high frequencies and wide at low frequencies, providing multi-resolution analysis that captures both short, high-frequency transients and long, low-frequency trends [56]. This adaptability produces scalograms that clearly illustrate how signal energy varies across time and frequency, offering a richer representation of fault signatures. These scalograms serve as informative inputs for convolutional neural networks (CNNs), which can automatically learn discriminative features, leading to more accurate and robust fault classification results. Figure 8 shows STFT and CWT plotted for the same conditions but for two different classes. It is observed that CWT scalograms provide a much more robust differentiation in time–frequency representation compared to STFT.

## 4. Experimental Setup, Data Acquisition, and Signal Preprocessing

**Figure 9:**
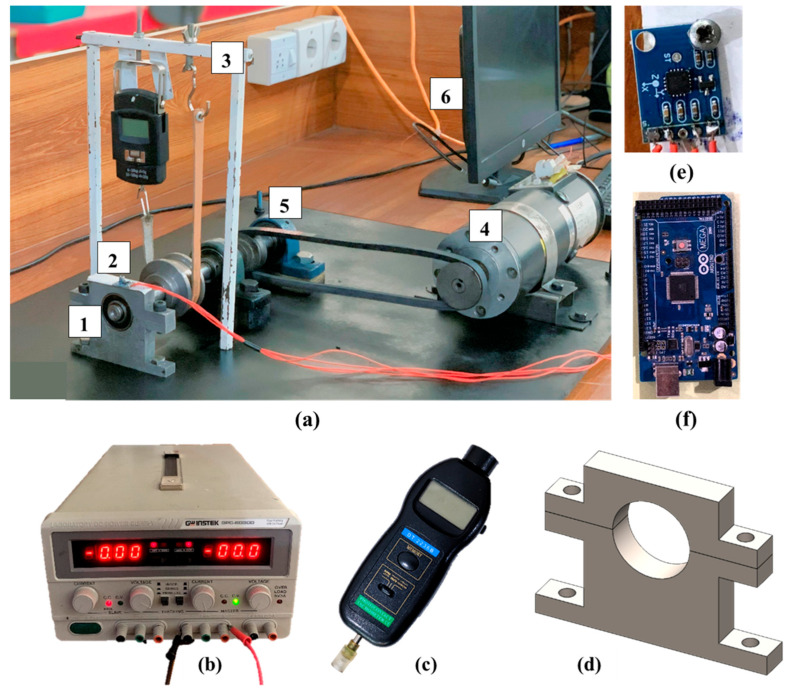
(**a**) Experimental setup: (1) test bearing, (2) ADXL 345 Sensor, (3) loading mechanism, (4) DC motor, (5) supporting bearing, and (6) connected computer. (**b**) DC power supply. (**c**) Tachometer DT-2236B. (**d**) Bearing housing. (**e**) Accelerometer sensor. (**f**) Arduino.

An in-house experimental setup is fabricated to extract data for bearing fault diagnosis. This setup consists of DC motors (4.5 Nm, 140 V, 75 A, 4000 RPM). Figure 9a shows the experimental setup. The rotating speed of the shaft is varied between 200 RPM and 600 RPM by varying the voltage through the GPC-6030D DC power supply (GW Instek, Suzhou, China, output watts 375 W, 0~60 V). Figure 9b shows the GPC-6030D DC power supply. The DC motor rotates the shaft with the help of a pulley belt system. A tachometer DT-2236B (range 2.5~99,999 RPM) is used to measure the rotating speed of the shaft at the start of the experiment, either by touch mode or optical mode, as shown in Figure 9c. The UCP-204 bearing housing unit is used to support the shaft at one end. The deep groove ball bearing 6203 that has dimensions of 12 mm in width, 40 mm in outer diameter, and 17 mm in bore diameter is selected as a test bearing and is placed on the other side of the shaft in a fabricated bearing housing. Figure 9d shows the SolidWorks (2024) model of bearing housing. The belt pulley system and modified bearing housing overcome the height adjustment issue between the motor shaft and the test bearing after each experiment cycle. The load on a rotating shaft varies between 0 kg and 0.75 kg using a belt and wing nut mechanism with the SrO VIRGO-A08-HANGING scale. The loading mechanism and DC power supply help to create varying load and speed conditions for the test bearings.

**Figure 10:**
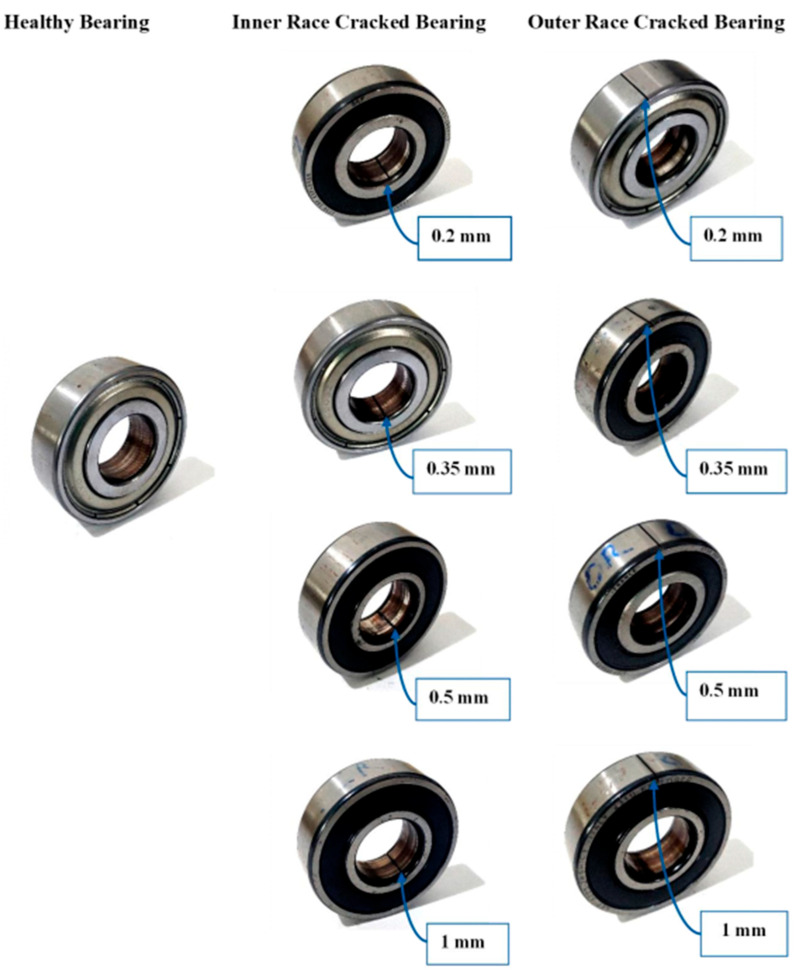
Bearing utilized for data extraction.

**Figure 11:**
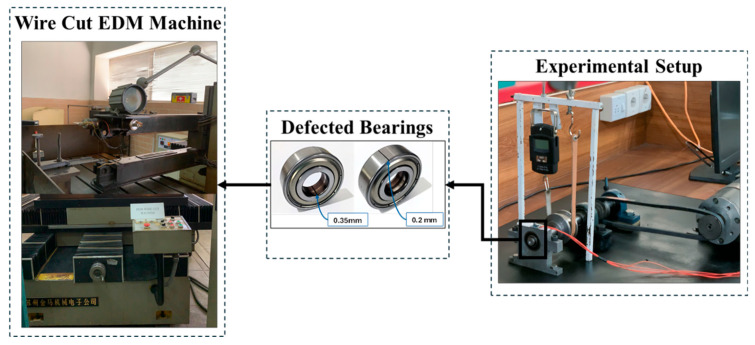
Wire cut electrical discharge machining (EDM) for defect generation.

Collecting bearings with defects from industries is a time-consuming and challenging task. Alternatively, artificial faults can be introduced on the inner and outer surfaces of the bearing for fault diagnostic purposes. Thus, nine deep groove 6203 ball bearings are selected. Bearings used for the present study are listed in Figure 10. One bearing is considered healthy with no faults. For the other eight bearings, the wire electrical discharge machining (WEDM) technique is used to generate 0.2 mm, 0.35 mm, 0.5 mm, and 1.0 mm cracks on the inner and outer raceways of the bearings, as shown in Figure 11. Thus, it leads to a total of nine classes: healthy, inner race 0.2 mm, inner race 0.35 mm, inner race 0.5 mm, inner race 1.0 mm, outer race 0.2 mm, outer race 0.35 mm, outer race 0.5 mm, and outer race 1.0 mm. Research has been performed. The crack size range was chosen based on the previous literature [33,57] and experimental feasibility, ensuring that the faults were both representative of real-world degradation levels and practically achievable in a controlled laboratory setting.

### 4.1. Data Acquisition

Vibration data is acquired from all bearings listed above at four different loads: no load, 0.2 kg, 0.5 kg, and 0.75 kg load. The SrO VIRGO-A08-HANGING scale is used to visualize the applied load. For each applied load, there are four different speeds: 200, 300, 400, and 600 RPM. According to the literature, previous research has been conducted on varying loads and with constant speed or vice versa. Based on the motor specification, a safe and suitable range of loads and speeds has been selected. Table 1 shows experiment scenarios for healthy bearing; similar scenarios are followed for the eight faculty classes. This resulted in a total of 576 dataset files, as data was collected for both the x and z axes (directions that are perpendicular to the bearings’ axis of rotation). Each experiment was repeated twice by disassembling and reassembling the bearing housing to minimize the potential for human error and to ensure a more robust and comprehensive dataset.

The vibration data on the x-axis and z-axis are collected at a sampling frequency of 10,000 Hz for 10 s used the ADXL345 accelerometer (vibration sensor), which is a cheap, thin, three-axis, 13-bit, high-resolution accelerometer, as shown in Figure 9e. The data acquisition rate is chosen based on the Nyquist theorem to avoid the aliasing effect or loss of important information in the collected signals. Arduino Mega 2560 is utilized to transfer data from the sensor to the computer interface for real-time monitoring and analysis, as shown in Figure 9f. The Simulink model reads this real-time data from the input source and stores it in the MATLAB (2024) workspace. This data is saved on a computer drive with appropriate names assigned according to the bearing type, load, and rotating speed at which they are tested.

### 4.2. Signal Preprocessing

**Figure 12:**
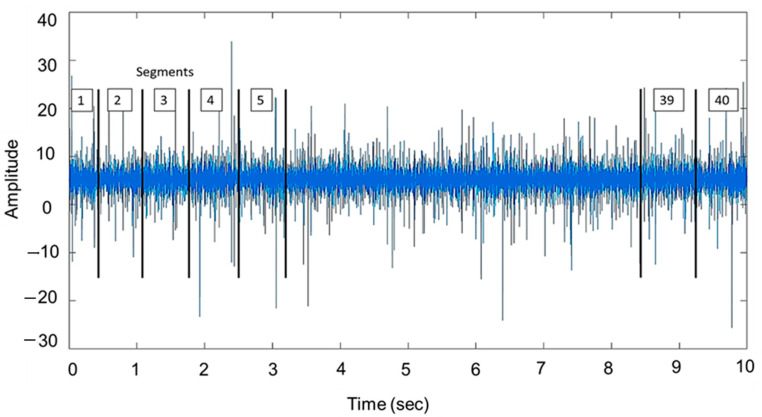
Signal segmentation into 40 segments.

By applying several approaches to the raw data, such as signal segmentation, normalization, and feature extraction, preprocessing aims to improve machine learning training [35]. In many studies, researchers have segmented the raw signal into smaller parts, either to extract more features from data or to divide it into training and testing datasets, when there is a limited amount of raw data signals available [58,59]. In this study, various types and severities of bearing faults were analyzed. Each dataset has 100,000 data points with a sample frequency of 10,000 Hz for 10 s. Still, every measured signal is segmented into 40 segments of 2500 points each. This will increase the overall data size for training and testing, improve feature extraction, better model generalization, and lead to efficient processing, as dealing with smaller segments is faster compared to larger signals. After segmenting the raw signal and extracting the features from each measurement segment, a feature selection tool is used to choose the best features. Figure 12 shows the signal segmentation process.

## 5. Results and Discussion

### 5.1. Machine Learning

**Figure 13:**
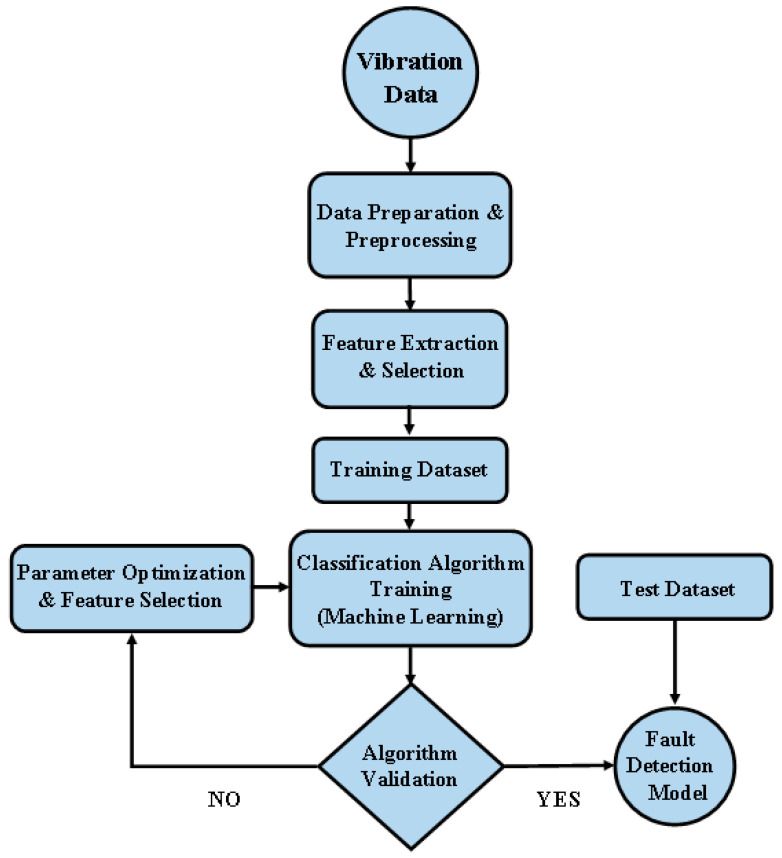
Machine learning workflow.

Figure 13 shows the general workflow of rotating machines and bearing failure diagnostics using machine learning. A variety of sensor devices are used to gather vibration data from both healthy and defective bearings. This raw data undergoes preprocessing to remove noise and artifacts, and features are then extracted to represent the underlying patterns indicative of different bearing conditions. The most valuable features for the diagnosis job are found using feature selection approaches once features have been retrieved. Different machine learning models are then trained and validated using these chosen characteristics. Until an acceptable degree of accuracy is attained, the training procedure is repeated multiple times. Lastly, the general performance of the trained model is assessed using test data that has not yet been observed. The machine learning model with the highest accuracy on both training and testing sets is mainly preferred for deployment in real-world applications.

#### 5.1.1. Feature Extraction and Selection

Feature engineering has a significant impact on algorithm performance for machine learning tasks. By finding underlying patterns, feature engineering increases the accuracy of machine learning algorithms. Various domain characteristics, including frequency domain, time domain, and time–frequency domain features, are retrieved from the raw signals.

The data used in this study is first separated into training and testing data. A total of 10% (1724 instances) of the entire data is kept as unseen for model testing, while the remaining 90% (17,240 instances) of data is used for training. Following the extraction of statistical characteristics, the data is separated into training and testing datasets. In total, 35 features, comprising different time domain, frequency domain, and time–frequency domain features, were manually extracted. For the time domain, mean, integrated EMG, standard deviation, skewness, kurtosis, peak to peak, root mean square (rms), crest factor, shape factor, impulse factor, margin factor, energy, zero crossing rate (ZCR), mean absolute deviation, variance, minimum, and maximum are evaluated. For the frequency domain, the following parameters are extracted: spectral centroid, spectral spread, spectral entropy, spectral flux, dominant frequency, frequency variance, band power, mean frequency, median frequency, bandwidth, spectral flatness, spectral skewness, spectral kurtosis, and peak frequency. For the time–frequency domain, scalogram-based features like SK mean, SK standard deviation, SK skewness, and SK kurtosis are extracted.

**Figure 14:**
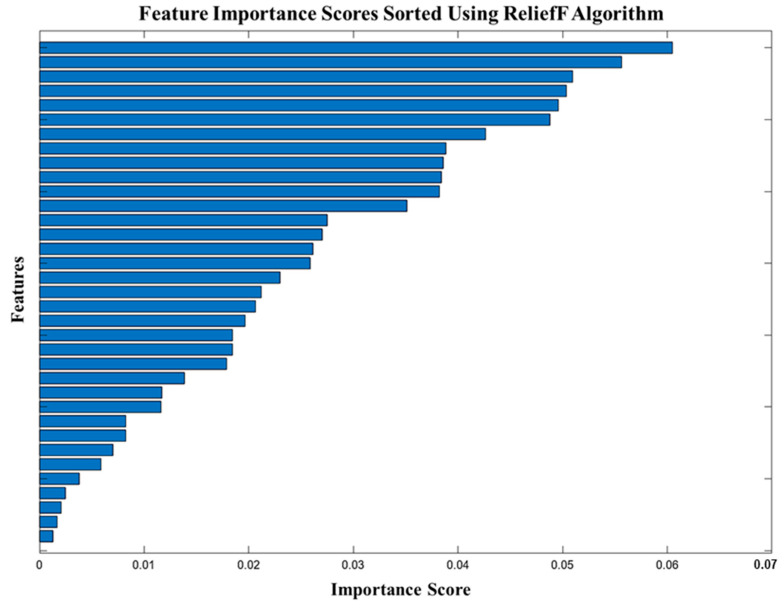
Feature importance score.

After extracting the features, the next step is to perform feature selection. One of the crucial phases in machine learning for bearing failure diagnostics is feature selection, which involves choosing the most relevant and informative aspects from the dataset while eliminating redundant or unnecessary features. Thus, feature selection improves the model’s efficiency, lowers the likelihood of overfitting, and facilitates interpretation without sacrificing accuracy. For feature selection, researchers have used various methods like ANOVA [60], chi-square [61], and ReliefF [62]. As the ReliefF method can tolerate noisy and incomplete data, it is especially selected for feature selection in this study. This makes it resilient for real-world applications such as vibration data analysis in fault diagnostics. Unlike other methods that evaluate features independently, ReliefF effectively captures interactions between features, making it ideal for complex datasets. Additionally, its flexibility in dealing with both binary and multiclass problems further justifies its use over other traditional methods. Importance scores of all features are calculated using the ReliefF algorithm, and those that are negatively impacting the machine learning model’s accuracy are discarded. A scatter plot is plotted to show the importance of all 35 features, as shown in Figure 14. Since there are no negatively impacting features, all 35 features are selected for training the machine learning models. Machine learning methods such as decision trees, discriminant analysis, logistic regression, naive Bayes, support vector machines, k-nearest neighbors, kernel approximation, ensembles, and neural network models are then trained and tested using these chosen features.

#### 5.1.2. Machine Learning Results

In this study, two separate machine learning studies have been performed for bearing fault diagnosis. The first study focuses on three class classification problems, where the bearings are divided into three groups: inner race fault, outer race fault, and healthy bearing. In the second study, the classes have been expanded to nine distinct conditions. There were cracks of different widths in both the inner and outer races, along with a variety of fault severities and healthy bearings. For the inner and outer races, cracks measuring 0.2 mm, 0.35 mm, 0.5 mm, and 1.0 mm are specifically regarded as distinct classes to evaluate the categorization of various defect severity levels. Ten-fold cross-validation is used for both investigations to guarantee the robustness and reliability of the models. In this study, the machine learning (ML) models were trained using MATLAB’s default hyperparameters available in the Classification Learner Toolbox. These default settings are well-calibrated for general classification tasks and provide a strong baseline for comparative performance evaluation.

**Figure 15:**
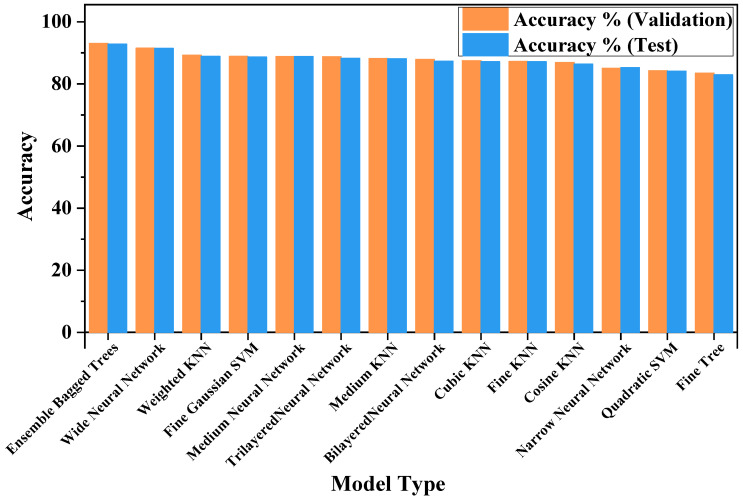
Validation and test performance of various algorithms for the three classes problem.

**Figure 16:**
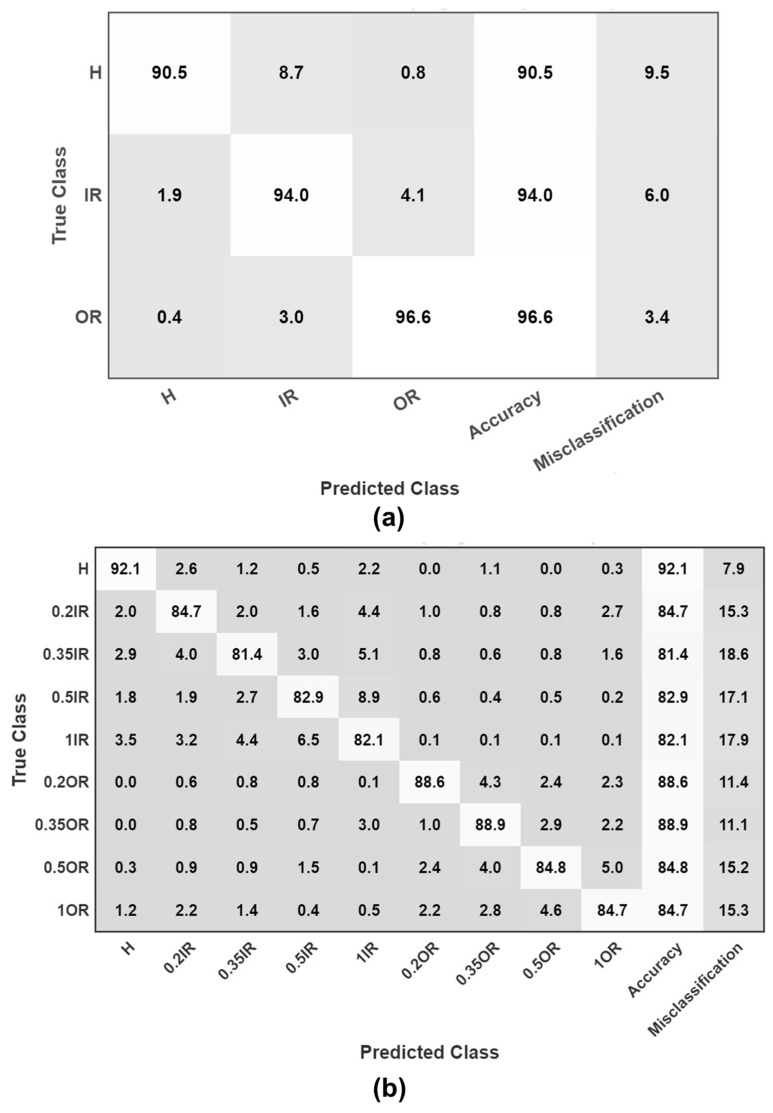
(**a**) Confusion matrix for ensemble bagged trees on a three-class problem. (**b**) Confusion matrix for ensemble bagged trees on a nine-class problem.

Figure 15 shows the results of various machine learning models used for three-class diagnosis. The ensemble bagged trees model achieved a maximum accuracy of 93.04% during validation and 92.79% on the test dataset, indicating its strong generalization capabilities. Figure 16a shows the confusion matrix of the ensemble bagged trees model. Among the neural network models, the wide neural network has the best performance accuracy of 91.55% on the validation set and 91.48% on the test dataset. K-nearest neighbor (KNN) and support vector machine (SVM) achieved competitive outcomes, but their overall accuracy is slightly lower than that of ensemble and neural network models.

**Figure 17:**
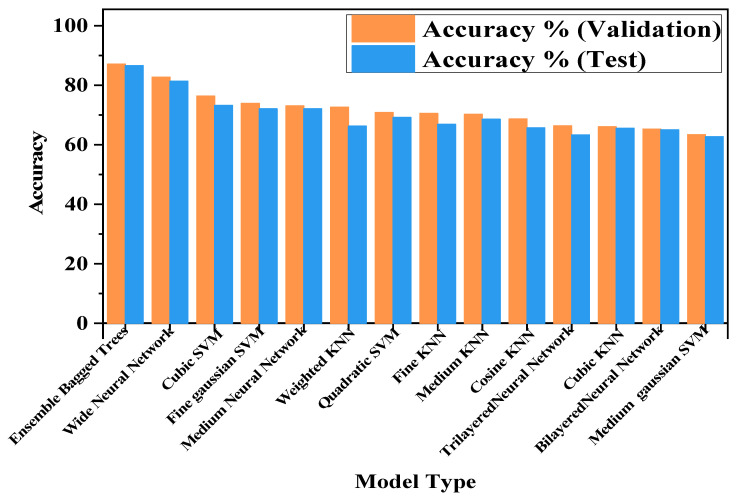
Validation and test performance of various algorithms for the nine-class problem.

For the more challenging nine-class classification task, accuracy drops slightly compared to the three-class problem. This is because the model now has to predict similar classes of damage with varying severity levels, which introduces minor differences and increases the complexity for the models. The ensemble bagged trees model again performed best, achieving 87.13% accuracy on validation and 86.63% on the test dataset. Figure 16b is the confusion matrix of the ensemble bagged trees model for the nine-class case. The neural network showed slightly decreased performance, with a wide neural network model achieving 82.74% accuracy on the validation set and 81.40% on the test dataset. SVM and KNN models struggled with additional complexity, showing significantly lower accuracy compared to the three-class classification. The overall outcomes of many machine learning models for the categorization of nine classes are shown in Figure 17.

Table 2 shows the precision, recall, and F1 score evaluation metrics of machine learning for both three- and nine-class scenarios. The performance evaluation results demonstrate that the developed model achieves strong classification accuracy across all bearing fault categories. In the nine-class scenario, the average precision, recall, and F1-score are 85.65%, 85.58%, and 85.59%, respectively. These values indicate that the model maintains balanced performance between correctly identifying true positives and minimizing false classifications. Among the classes, the healthy (H) and outer race faults (OR) show relatively higher precision and recall, suggesting that these fault types exhibit more distinguishable signal patterns. In contrast, the inner race fault classes, particularly IR_1, show slightly lower scores, likely due to overlapping spectral characteristics and signal similarities among different fault severities, which make them more challenging to differentiate.

When the classes are grouped into a simplified three-class problem (H, IR, OR), the model’s performance improves significantly, achieving an average precision of 93.87%, recall of 93.70%, and F1-score of 93.72%. This improvement indicates that the model is highly effective in identifying the general fault category but faces some difficulty in distinguishing between fine-grained fault severities. Overall, the results validate the robustness of the proposed approach for bearing fault diagnosis, with excellent general fault detection capability and reasonably strong fine-level classification performance.

While the machine learning models demonstrated better performance for the three-class classification problem, the solution has limited applicability in practical scenarios. In the nine-class classification problem, traditional machine learning models like KNN and SVM struggled to maintain accuracy due to the similar types of damage of different severity levels. This indicates that machine learning models may not be well-suited for capturing minor differences in the data, such as varying crack size in inner and outer races. In addition, traditional machine learning requires manual feature engineering and parameter tuning, which can be time-consuming and may still miss important patterns in the data. To overcome these challenges, the autonomous learning characteristics of deep learning techniques can be employed. Unlike machine learning, deep learning methods can automatically learn complex generalized features from the original data, reducing the need for manual feature extraction, making them well-suited for high-dimensional and complex input [63,64]. This use of deep learning is expected to increase classification performance, particularly for the nine-class classification task, where the goal is to generalize the model for varying levels of fault severity in the presence of load and speed fluctuations. Statistical validation was also performed using 10-fold cross-validation and 95% confidence intervals for all accuracy results. The machine learning models achieved 85.69% (95% CI: 83.47–87.91%) and 93.7% (95% CI: 91.0–96.4%), respectively. These findings confirm that the results are statistically consistent and experimentally reliable, reflecting the robustness of the proposed approach.

### 5.2. Deep Learning

**Figure 18:**
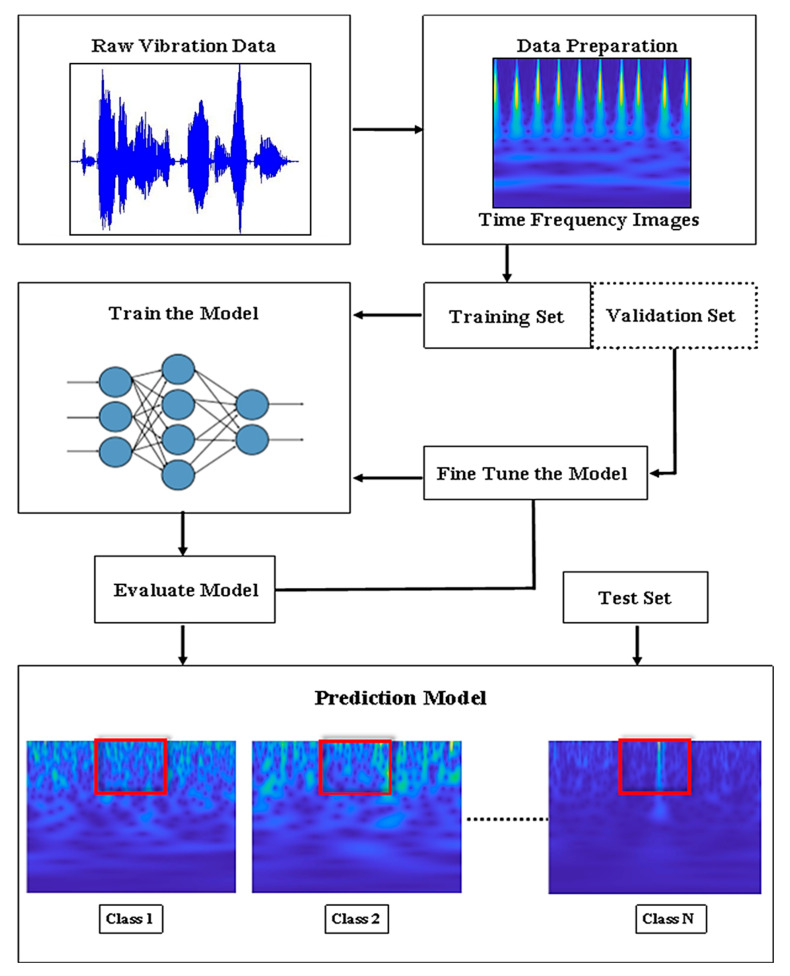
Workflow of deep learning.

Deep learning has emerged as one of the most important areas of research and has been applied to many other sectors [65,66,67,68]. CNN is the most used deep learning algorithm for image processing applications such as object identification [69], face recognition [70], and image classification [71]. Figure 18 shows the general workflow of deep learning for fault diagnosis. The continuous wavelet transform function is used to convert the raw vibration data into time–frequency pictures. The deep learning model can easily recognize distinct patterns for various fault kinds, as these images aid in visualizing the vibration signal’s frequency content over time. After that, the data is divided into sets for training, testing, and validation. The deep neural network is trained using the training set. Due to its inherent design, the neural network automatically extracts significant features from the pictures, removing the requirement for human feature engineering. After initial training, the model is fine-tuned using a validation set to optimize its parameters, improving its generalization to new data. Finally, the model is evaluated on the test set, and it is deployed for fault classification, distinguishing between various fault classes.

For this study, the deep learning process began by generating scalograms (RGB images) using a continuous wavelet transform function from the raw vibration data, which consisted of a total of 100,000 data points. A corresponding scalogram is produced for every segment, capturing the vibration signal’s time–frequency representation. After that, 90% of these scalograms are used for training, while the remaining 10% are used for testing. Training scalograms are used for training the convolutional neural network (CNN) with the architecture shown in Table 3. The scalograms have different time–frequency distributions for different classes, as annotated in Figure 18. The CNN model differentiates between fault classes by learning from the time–frequency patterns in the scalograms of vibration signals. Although these variations are often too subtle to be noticed by the human eye, the CNN can detect them by sliding filters across the image and identifying meaningful patterns in the data. Through this process, the model gradually learns the distinctive features of each class, allowing it to accurately recognize and classify faults based on their unique time–frequency characteristics. The CNN architecture used in this study was developed through a step-by-step empirical process, starting from a simple baseline model inspired by previously published literature on vibration-based fault diagnosis using time–frequency representations. Initially, a minimal CNN configuration with a single convolutional layer was implemented to evaluate baseline performance. Based on the initial results, we iteratively refined the number of convolutional layers, filter sizes, and learning rate using a trial-and-improvement approach, guided by the model’s training stability and validation accuracy. This iterative tuning led to an optimized configuration that achieved consistent and high classification performance across all experiments. The primary objective of this study is not to optimize or fine-tune the CNN architecture but rather to demonstrate the applicability and effectiveness of a simple CNN model for fault classification under varying load and speed conditions. It is well-recognized that CNNs contain many hyperparameters and architectural variations, and extensive optimization can lead to numerous model configurations. However, since the focus of this research is on the methodological application of AI for mechanical fault diagnosis, rather than developing an idealized or highly optimized CNN, a baseline CNN structure was employed. CNN’s input layer takes RGB pictures that are 224 × 224 × 3 in size. A convolutional layer with 16 3 × 3 filters comes next. By standardizing the activations throughout the micro-batch, batch normalization is used to stabilize and expedite training. The max-pooling layer (size 2 × 2 with stride 2) is used to lower the scalograms dimension and capture the most noticeable characteristics, while the ReLU activation layer is used to induce non-linearity. The fully connected layers map these features with the output size corresponding to the number of classes, either three or nine. A SoftMax layer for multiclass classification and a classification layer to assign labels based on prediction probabilities are attached at the end for predictions. The Adam optimizer was used to train the model, and the learning rate schedule was set to drop by 0.2 every five epochs. With a tiny batch size of 64, we trained the model for one, three, five, and six epochs. To guarantee reliable performance across various splits, we tracked the training process and validated the model using the validation data at each epoch. The continuous wavelet transform (CWT) was employed as the wavelet function to generate scalograms from vibration signals, providing a rich time–frequency representation suitable for CNN-based feature extraction. The dataset was divided into 90% training and 10% testing to ensure a fair and representative evaluation of model performance. The CNN was trained using the Adam optimizer with the following parameters: LearnRateSchedule = piecewise, LearnRateDropFactor = 0.2, LearnRateDropPeriod = 5, MaxEpochs = 6, MiniBatchSize = 64, and Shuffle = every-epoch. The data is validated to monitor performance during training.

In this approach, 10-fold cross-validation is implemented, so the dataset was randomly shuffled and then divided into 10 equal subsets. During each iteration, nine folds were used for training, while the remaining fold was used for testing. This process was repeated 10 times so that every data segment was used once for testing.

#### Deep Learning Results

Like machine learning, separate analysis has been carried out for both the three-class and nine-class classification tasks using deep learning. For the three-class classification, CNN was trained to identify healthy bearings from inner and outer race defective bearings. The nine-class study expanded the classification to capture different severities of cracks in both the inner and outer races of the bearing. The output layer of the fully connected layer was modified to reflect the number of classes—three for the first study and nine for the second—and this was the only modification made to the CNN model between the two tasks.

**Figure 19:**
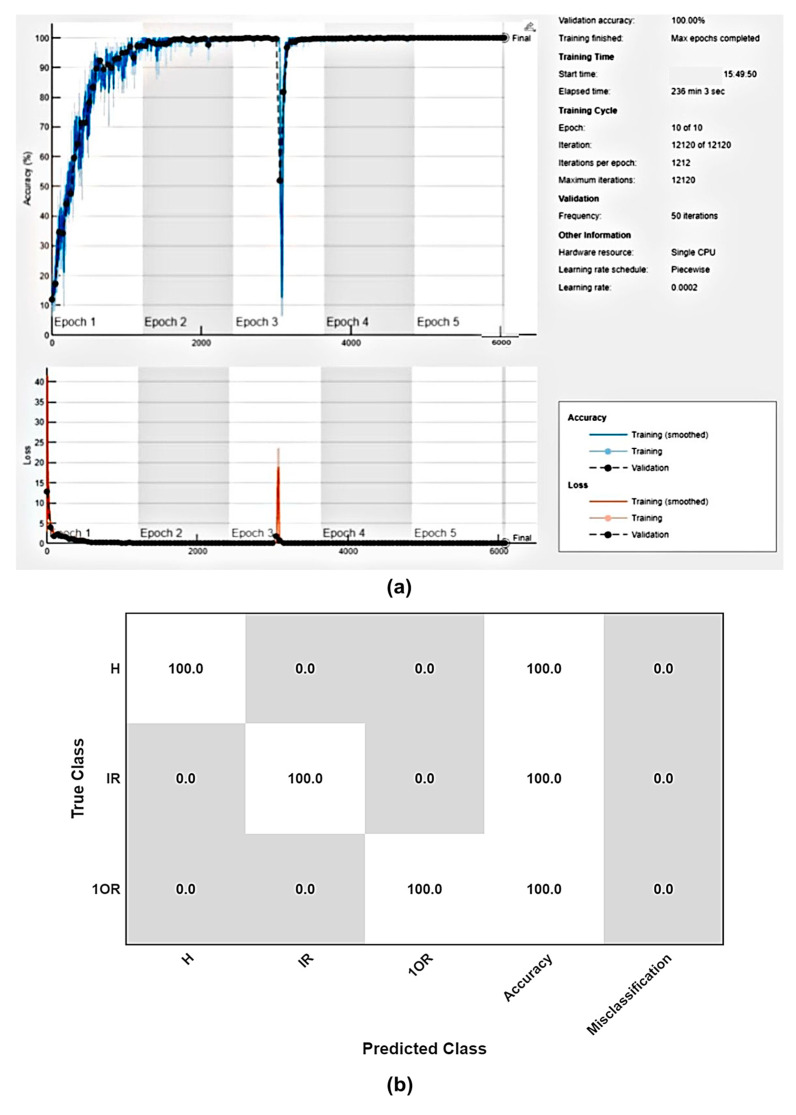
Plot of (**a**) training and validation accuracy and loss. (**b**) Confusion matrix for three-class problem.

For the three-class classification problem, the CNN model demonstrated exceptional performance, achieving 99.27% training and 99.24% testing accuracy after just one epoch. As the number of epochs increased, the accuracy continued to improve to 99.41% for three epochs and perfect 100% training and testing accuracy after five epochs. Figure 19a shows the training progress for the CNN three-class classification study. It can be seen that with the increase in epoch number, accuracy increases, and correspondingly, the validation loss decreases. To address concerns about overfitting and reproducibility, the 90% training dataset was divided into training and validation subsets (70–30 split), and model performance was continuously monitored using MATLAB’s training-progress plots. The validation accuracy (black dots) closely followed the training accuracy curve (blue line) with minimal deviation, confirming that the model was not overfitting. Additionally, no significant gap was observed between training and validation loss values, further supporting the model’s generalization capability. This remarkable performance highlights the CNN’s ability to effectively learn and generalize from the data, with minimal training. Figure 19b shows the training confusion matrix after five epochs.

**Figure 20:**
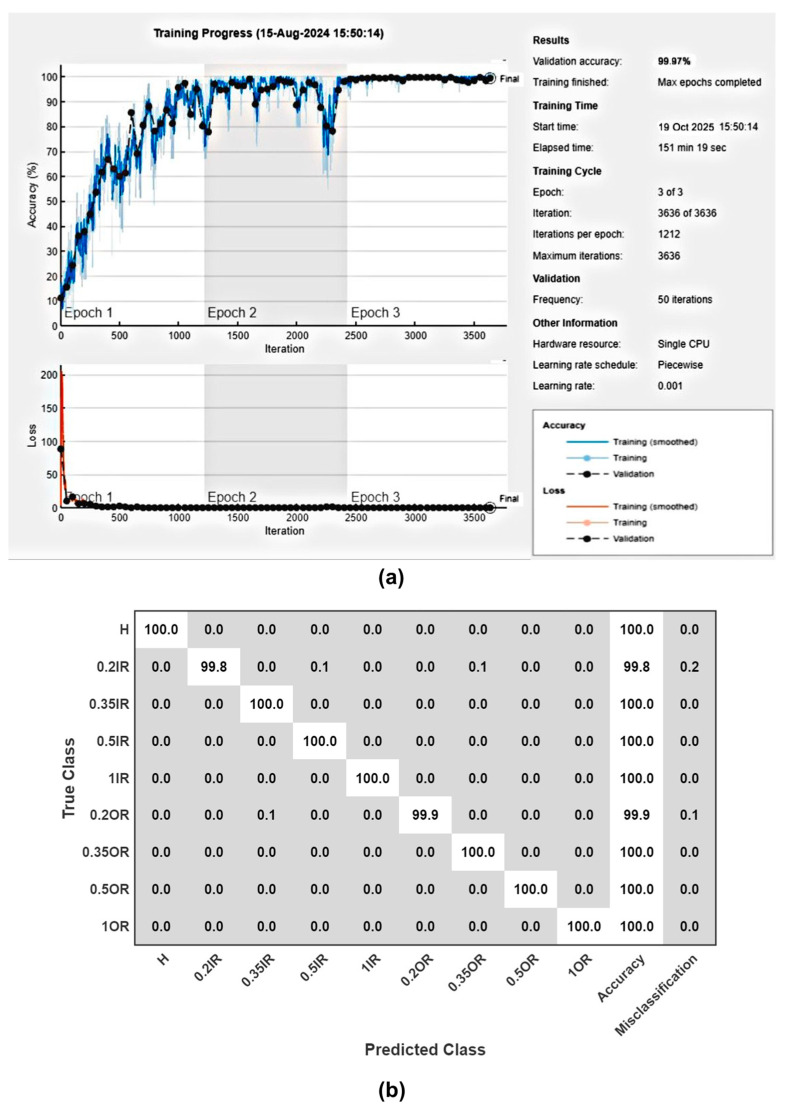
Plot of (**a**) training and validation accuracy and loss. (**b**) Confusion matrix for nine-class problem.

In the more challenging task, CNN also performed admirably. However, the added complexity of predicting across nine fault categories resulted in slightly lower initial accuracy. After one epoch, the model achieved 98.87% training accuracy and 98.74% testing accuracy. With additional epochs, accuracy improved, reaching 99.2% for three epochs and 99.74% for five epochs. Perfect accuracy of 100% is achieved on both training and testing sets for six epochs. Figure 20a shows the training and validation curves of a nine-class classification problem. These results demonstrate the robustness of the deep learning approach, which can maintain high levels of accuracy even for complex classification tasks. Figure 20b shows the training confusion matrix after six epochs.

Table 4 shows the precision, recall, and F1 score evaluation metrics of machine learning for both three- and nine-class scenarios. Deep learning results indicate exceptional model performance in both fine-grained (nine-class) and simplified (three-class) bearing fault classification scenarios. In the nine-class setup, the model achieved near-perfect metrics, with macro precision, recall, and F1 score of 99.97%, and an overall accuracy of approximately 99.97%. Each class, representing different fault severities of inner and outer race defects as well as the healthy state, exhibits precision, recall, and F1 scores above 99.8%, demonstrating the model’s outstanding ability to accurately detect and distinguish even subtle fault variations. This suggests that the features extracted are highly discriminative and that the classifier generalizes extremely well to unseen data without significant misclassification.

Similarly, in the three-class configuration (H, IR, OR), the model achieved a perfect 100% precision, recall, and F1 score for all categories, reflecting flawless classification between healthy, inner race, and outer race conditions. This level of accuracy highlights the strong robustness, stability, and adaptability of the model in handling different fault types and complexities. Overall, these results confirm the superior diagnostic capability of the proposed approach, effectively capturing discriminative fault features and providing reliable, high-confidence predictions across all bearing fault categories. Statistical validation was also performed using 10-fold cross-validation and 95% confidence intervals for all accuracy results. The deep learning model achieved an accuracy of 99.97% (95% CI: 99.85–100%) for the nine-class problem and 100% (95% CI: 100–100%) for the three-class case.

### 5.3. Comparison of Machine Learning with Deep Learning

The results for both machine learning and deep learning studies highlight key differences in their performance and applicability for bearing fault diagnosis. Machine learning models like ensemble bagged trees, neural networks, KNN, and SVM showed satisfactory results for three-class classification, but CNN outshone them, particularly as the complexity of the classification task increased.

For three-class classification, both machine learning and deep learning approaches performed well. The ensemble bagged trees model from machine learning achieved the maximum validation accuracy of 93.04% and test accuracy of 92.79%, indicating its strong generalization capability. The wide neural network achieved 91.55% validation accuracy, followed by KNN and SVM with validation accuracy of 89.2% and 88.9%, respectively. While these results are impressive, they were still surpassed by a deep learning CNN model, which achieved 99.27% validation accuracy and 99.24% testing accuracy after just one epoch. With additional epochs, the CNN quickly converged to 100% accuracy on both the training and test sets. This showcases CNN’s superior ability to learn complex features from the data, even with minimal training. Using CNN eliminates the need for manual feature engineering, a necessity in traditional machine learning algorithms. Moreover, the CNN achieved near-perfect accuracy across all epochs, indicating strong generalizations without risk of overfitting, as an increase in epoch may also result in overfitting.

As the classification task became more complex, with the introduction of varying crack sizes in both inner and outer races (nine-class problem), the performance of the machine learning model began to decline. The ensemble bagged trees model achieved 87.13% validation accuracy, while the wide neural network managed to achieve 82.74% validation accuracy. Other machine learning models, such as SVM and KNN, struggled to maintain similar performance levels, with significantly lower accuracies. As the cracks in the inner race and outer race are closely related, with sizes like 0.2 mm and 0.35 mm, the model occasionally confuses one inner race defect with another, which is reasonable given their similarities. Importantly, the model does not confuse inner race defects with outer race defects, highlighting its ability to differentiate between distinct fault types correctly. This shows that while it may struggle with fine differences within the same class, it maintains a firm grasp of the broader distinctions between different fault types. In contrast, deep learning demonstrated its true strength in nine-class classification. Although the initial CNN performance after one epoch was slightly lower than the three-class classification, the model still achieved 98.87% validation accuracy and 98.74% testing accuracy. With additional epochs, CNN’s performance improved, reaching 99.74% accuracy after five epochs and finally achieving perfect 100% accuracy, for both training and testing sets, after six epochs.

Statistical validation was also performed using 10-fold cross-validation and 95% confidence intervals for all accuracy results. The deep learning model achieved an accuracy of 99.97% (95% CI: 99.85–100%) for the nine-class problem and 100% (95% CI: 100–100%) for the three-class case. In comparison, the machine learning models achieved 85.69% (95% CI: 83.47–87.91%) and 93.7% (95% CI: 91.0–96.4%), respectively. These findings confirm that the results are statistically consistent and experimentally reliable, reflecting the robustness of the proposed approach.

Training time is a critical consideration when comparing machine learning and deep learning models [72], especially in industrial applications where efficiency and computational resources are key factors. In this study, the CNN model required approximately 195 min to train for the three-class classification task, while all the machine learning (ML) algorithms combined took about 163.4 min. For the more complex nine-class classification task, CNN training took around 257 min, compared to 216.48 min for the ML models. It is important to note that these times were recorded on a standard CPU without GPU acceleration. Deep learning models, particularly CNNs, are computationally intensive due to their large number of parameters and iterative backpropagation operations. However, this longer training time is often justified by the significantly higher accuracy and robustness observed in the results.

When executed on a dedicated GPU (NVIDIA RTX 3060), the training process was drastically faster, requiring only 52 min for the three-class model and approximately 1 h and 28 s for the nine-class model. This demonstrates the substantial computational advantage of GPU acceleration for deep learning. Moreover, once trained, the prediction phase of the CNN model is extremely fast, enabling real-time or near-real-time fault diagnosis, which is crucial for practical industrial applications.

### 5.4. Comparison with Published Literature

To evaluate the novelty of this work, the results are compared with those recently published in the literature. Table 5 provides a comparison of the published literature and its contributions with the present study.

## 6. Conclusions and Future Recommendations

This paper presents a comprehensive study of the application of deep learning (DL) techniques and machine learning (ML) algorithms for diagnosing bearing failures. The key contribution lies in the comparative evaluation of traditional machine learning (ML) and deep learning (DL) techniques for bearing fault diagnosis under simultaneously varying load and speed conditions, which closely reflects real-world industrial environments. With the use of wire cut electro-discharge machining (WEDM), faults were created on the bearing’s inner and outer races. To imitate real-world conditions, a thorough experimental setup was created, and features in the time, frequency, and time–frequency domains were extracted from the raw vibration data. Machine learning models such as ensemble bagged trees, neural networks, SVM, KNN, decision trees, and many others were implemented after feature selection through the ReliefF algorithm. These models performed reasonably well, with the ensemble and neural networks achieving the highest accuracy of 87.13% and 82.74%, respectively, in the nine-class classification task. However, the complexity and variability of the bearing fault data proved challenging for traditional machine learning models, especially under simultaneous variations in load and speed conditions.

Superior performance was shown by the deep learning, convolutional neural network (CNN) model. With its ability to automatically extract features from time–frequency images, the CNN model achieved near-perfect classification accuracy (100%) after only six epochs. This highlights the potential of deep learning to handle complex and high-dimensional data more effectively than traditional machine learning models, particularly in scenarios with multiple fault types and varying operating conditions. In terms of simplicity and computational efficiency, traditional machine learning models such as SVM, KNN, and ensemble bagged trees proved advantageous due to their lower training times and smaller data requirements. These models are easier to implement and are suitable for real-time fault detection scenarios. In contrast, the deep learning (DL) approach requires greater computational power and training time, but offers enhanced reliability and accuracy by automatically extracting discriminative features from time–frequency representations. Overall, the findings show that CNN is a more reliable and expandable method for diagnosing faults in dynamic settings. This study shows that employing artificial intelligence to diagnose bearing problems in rotating machines while varying load and speed simultaneously is feasible and effective. The high accuracy of deep learning paves the way for an autonomous fault diagnosis system that can significantly enhance the performance and reliability of rotating machines.

Despite the promising results, this study has certain limitations that should be acknowledged. The experiments were conducted using a single bearing type (6203) under laboratory-scale load and speed conditions, which, while controlled, may not fully capture the complexities of industrial environments. Additionally, only single localized faults were introduced on the inner and outer races, and compound or mixed faults were not considered. These factors may influence the model’s ability to generalize to different bearing geometries, materials, or fault combinations. Acknowledging these constraints helps clarify the scope of this work while highlighting opportunities for future extensions, such as validating the models on different bearing types, load profiles, and compound fault scenarios to further enhance their industrial applicability and robustness.

Future research should focus on making machine learning (ML) and deep learning (DL) models more reliable, flexible, and applicable for real-world fault diagnosis in rotating machinery. A key next step is to use more diverse datasets that cover a broader range of operating conditions, load variations, and fault types. Expanding the data in this way will help these models generalize better and remain effective outside controlled laboratory environments. Another important direction is the diagnosis of combined or compound faults, where several issues may co-occur. This reflects how machinery behaves in actual industrial settings and remains a challenging area of research. Exploring hybrid approaches that combine the strengths of both ML and DL techniques could also improve diagnostic accuracy and make the models more interpretable. In the future, integrating these models into real-time monitoring systems will be an exciting step forward. Real-time AI-based (IOT-integrated) diagnosis can detect early signs of failure, allowing quicker interventions and helping to minimize downtime. Finally, collaboration with industry partners will be essential to test and validate the proposed models on real machines, ensuring that the developed techniques move beyond the lab and deliver practical value in industrial applications.

## Figures and Tables

**Table 1:** Experiment scenarios of load and speed variations for healthy bearing.

Bearing Type	Load Variation (kg)	Speed Variation (RPM)
Healthy bearing	0	200
		300
		400
		600
	0.2	200
		300
		400
		600
	0.5	200
		300
		400
		600
	0.75	200
		300
		400
		600

**Table 2:** Precision, recall, and F1 score evaluation metric for machine learning cases.

Class	Precision %	Recall %	F1 Score %
**Three-Class Classification**			
H	97.522	90.5	93.88
IR	88.931	94	91.395
OR	95.172	96.6	95.881
Average	93.87	93.70	93.72
**Nine-Class Classification**			
H	88.728	92.1	90.383
IR 0.2mm	83.94	84.7	84.32
IR 0.35mm	85.41	81.23	83.27
IR 0.5 mm	84.67	82.98	83.8
IR 1 mm	77.16	82.01	79.51
OR 0.2 mm	91.64	88.68	90.13
OR 0.35 mm	86.31	88.9	87.58
OR 0.5 mm	87.51	84.88	86.17
OR 1 mm	85.46	84.7	85.08
Average	85.65	85.58	85.59

**Table 3:** CNN architecture employed in this study.

Layer Name	Description
Image Input Layer	Input: 224 × 224 × 3 (size of RGB images)
Convolution 1	Convolution layer (3 × 3 filter, 16 filters,)
Batch Normalization	Normalizes the activations across the mini batch
ReLU Layer	Activation layer (rectified linear unit)
Max-Pooling 1	Max-pooling layer (2 × 2 filter with stride 2)
Fully Connected Layer	Fully connected layer with output size (depends on the number of classes: either three or nine)
SoftMax Layer	SoftMax function for multiclass classification
Classification Layer	Assigns the class labels based on the SoftMax output

**Table 4:** Precision, recall, and F1 score evaluation metric for deep learning cases.

Class	Precision %	Recall %	F1 Score %
**Three-Class Classification**			
H	100	100	100
IR	100	100	100
OR	100	100	100
Average	100	100	100
**Nine-Class Classification**			
H	100	100	100
IR 0.2 mm	99.8	99.88	99.8
IR 0.35 mm	100	100	100
IR 0.5 mm	100	100	100
IR 1 mm	100	100	100
OR 0.2 mm	99.9	99.9	99.9
OR 0.35 mm	100	100	100
OR 0.5 mm	100	100	100
OR 1 mm	100	100	100
Average	99.97	99.97	99.97

**Table 5:** Comparison of present study with published work.

Research	Faults Studied	Techniques	Contributions	Limitations
[37]	Ball fault. Inner ring fault. Outer ring fault. Combined fault (fault type not specified).	PCA (principal component analysis) for reducing data dimensionally. SVM (support vector machine) as a classifier. Analysis of variance (ANOVA) is used for feature selection.	Achieved 97.4% training accuracy. 90% test accuracy, indicating slight overfitting. Reasonable approach for variable speed conditions.	Constant load. Low severity levels.
[13]	Ball fault. Inner race fault. Outer race fault. Defects of diameter 0.007-inch, 0.014-inch, 0.021 inch.	Multiclass CNN and long short-term memory (MCNN-LSTM) method is used. CNN is used as a feature extractor. LSTM as a classifier.	Achieved 98.46% average test accuracy (10–15% higher than other machine learning and deep learning models). Works well for noisy environments.	Varying Speed. Constant load.
[73]	Ball fault. Inner race fault. Outer race fault. Defect of diameter from 0.007 inch to 0.04 inch.	Envelope analysis for featurization. Trained and tested multiple ML models in MATLAB like SVM, KNN, kernel naïve Bayes and many others.	KNN and decision tree achieved a perfect 100% accuracy. Naïve Bayes achieved 94.4% accuracy.	Varying speed. Constant load.
[39]	Ball fault. Inner race fault. Outer race fault. Defect of diameter from 0.007 inch to 0.04 inch. For self-designed testbed and for CWRU dataset.	Explainable AI (XAI)-based approach for bearing fault diagnosis. KNN classifier with additive Shapely additive explanations. Introducing Boruta for feature selection.	For the CWRU dataset 100% accuracy is achieved. For the in-house dataset, 97% accuracy is achieved.	Varying speed. Constant load.
[45]	Ball fault. Inner race fault. Outer race fault. CWRU and Paderborn datasets were used.	Traditional ML models like SVM, random forest, KNN, logistic regression and multi-layer perception (MLP) are used as classifiers. Fault Net, a CNN-based model, is used for deep learning classification.	97.77% classification accuracy for CWRU dataset and 98.8% for Paderborn University. Deep learning achieved 10–12% higher than machine learning models.	Varying speed. Constant load.
[46]	Ball fault. Inner ring fault. Outer ring fault. A total of 2368 dataset samples are available. Defect of diameter from 0.007 inch to 0.04 inch.	Gradient-class activation mapping (Grad-CAM) for feature selection. Convolutional neural network (CNN) as a classifier.	Training and testing accuracy of 100% achieved.	Varying speed. Constant load.
[43]	Ball fault. Inner race fault. Outer race fault. Damage diameter from 0.1778 mm to 0.7112 mm. CWRU and machinery fault prevention technology (MFPT) datasets are used.	CNN is used for feature extraction. SVM is used as a classifier.	The hybrid CNN-SVM model achieved 98.89% accuracy.	Varying speed. Constant load. Small data size.
[35]	Ball fault. Inner race fault. Outer race fault. Paderborn dataset is used.	Genetic algorithm for feature selection. Artificial neural network (ANN) for classification.	Model achieved 94.1% accuracy for vibration. 95% accuracy for motor current signal.	Varying speed. Constant load.
[12]	Outer race fault. The diameter and the depth of a hole are 0.5 mm and 0.5 mm, respectively. A scratch has a size of 5 mm in length and 0.5 mm in width and depth.	For machine learning, SVM, KNN, decision tree, naive Bayes, and random forest are considered. For deep learning CNN is used.	For SVM, 87.85% accuracy is achieved. For KNN, 83.04% accuracy is achieved. For CNN, 89.26% accuracy is achieved.	Constant speed. Varying load.
Present study	Inner race fault. Outer race fault. Crack of size 0.2 mm, 0.35 mm, 0.5 mm, 1 mm.	For ML, traditional ML algorithms like SVM, KNN, ensemble, NN, decision tree, and others are used. ReliefF algorithm is used for ML feature selection. For DL, CNN is used the architecture discussed above.	Among the traditional machine learning models, ensemble bagged trees and neural networks achieved the highest accuracies of 87.13% and 82.74%, respectively, for the complex nine-class classification problem, while SVM and KNN maintained accuracies above 75%. The CNN model outperformed all others, reaching 100% accuracy after six epochs. In addition to accuracy, performance was further validated using precision, recall, and F1 score metrics. The machine learning models achieved an average precision of 85.65%, recall of 85.58%, and F1 score of 85.59%, while the CNN achieved near-perfect results with all three metrics averaging 99.97%. These results confirm that CNN offers superior feature extraction and classification capability, making it highly effective for bearing fault diagnosis under varying load and speed conditions.	Varying load. Varying speed. Large data size considered to overcome previous studies’ limitations.

## Data Availability

The dataset can be provided upon reasonable request to the corresponding author.
